# Quantifying roadless areas and fragmentation in the context of wildlife-vehicle collision risk in Great Britain

**DOI:** 10.1038/s41598-026-36410-8

**Published:** 2026-02-18

**Authors:** Sarah Raymond, Elizabeth A. Chadwick, Sarah E. Perkins

**Affiliations:** The Sir Martin Evans Building, School of Biosciences, Cardiff University, Cardiff, CF10 3AX UK

**Keywords:** Wildlife-vehicle collisions, Roadless areas, Protected areas, Fragmentation, Ecology, Ecology, Environmental sciences

## Abstract

Roads cause indirect and direct effects for wildlife, including pollution, behavioural changes, wildlife mortality and habitat fragmentation. The ecological impacts of roads extend beyond their immediate vicinity, into ‘road effect zones’. Beyond road effect zones, ‘roadless areas’ can offer refuges from the ecological effects of roads. In this study, we quantify the number and distribution of roadless patches in Great Britain, their ecological status and their overlap with protected areas. Road effect zones applied 100, 500 and 1,000 m on either side of the road network revealed a highly fragmented landscape with 93,561, 29,164 and 6,138 roadless patches, covering 76.6%, 36.0% and 20.6% of terrestrial land, respectively. Roadless areas were smallest, on average, in England and largest in Scotland. For all road effect zones, more than 70% of patches were smaller than 1 km^2^. For the most commonly used road effect zone (1 km), more than 60% of roadless patches were smaller than the mean home range of common UK species, European badger *Meles meles* and red fox *Vulpes vulpes*, meaning they likely face a high risk of wildlife-vehicle collisions. Nearly half (47%) of roadless areas coincided with protected land and there was a weak but significant negative relationship between roadless patch size and ecological status. In conclusion, the landscape is fragmented into numerous, small roadless patches. We present roadless areas and their ecological features as an open access searchable map, constituting a tool for conservation practitioners and an important starting point for defragmentation campaigns.

**Supplementary Information:**

The online version contains supplementary material available at 10.1038/s41598-026-36410-8.

## Introduction

Roads are ubiquitous in terrestrial environments and globally cover 20% of land, fragmenting the remainder into almost 600,000 patches^1^. It is therefore unsurprising that roads have a wide range of reported impacts on wildlife, including reduced habitat connectivity as well as direct mortality from wildlife-vehicle collisions (WVCs)^2^. Indeed, WVCs are estimated to cause millions of animal mortalities on roads every year and are a leading cause of population decline and biodiversity loss^3,4^, with variable WVC-risk according to species’ traits^5,6^. Roads alter natural landscapes, producing anthropogenically modified environments that constitute ‘novel ecosystems’^7^, characterised by challenges and opportunities that would not previously have occurred^8^. Consequently, a comprehensive understanding of their environmental impacts is essential.

The ecological effects of roads on wildlife and the environment are not limited to the immediate vicinity of the road but extend outwards from the road with impacts commonly being reported with a 1 km buffer around roads (e.g.^1^) – known as the 'road effect zone’^9,10^. The spatial extent of the road effect zone is, in reality, dependent on the species of interest^11^, the habitat and terrain, and also the specific type of effect in focus (e.g. noise or light pollution, chemical emissions, road avoidance, WVCs)^1,12^. Soil contamination, for example, is relatively localised (30 m road effect zone;^13^), whilst the effects of roads on ecological traits and behaviour of large mammals and amphibians has been observed to extend from 600 to 1,000 m, respectively^14,15^. Beyond the road-effect zones are ‘roadless areas’: defined as patches of land unaffected by roads^1^. Roadless areas represent relatively undisturbed habitats and functioning ecosystems; they enhance connectivity of the landscape and provide barriers to invasive non-native species ingress^16,17^ and disease, including zoonotic spillover^18,19^. As well as serving as important terrestrial refugia they provide societal benefits by playing a role in regulating ecosystem services, for example, in soil protection, water retention and carbon sequestration^20,21^. Importantly, roadless areas may also protect some species, depending on the size, from the negative impacts of roads, acting as ‘road refuges’. For these reasons, roadless areas are posited to be synonymous with areas of high conservation priority^20^, as well as being a focus of future conservation efforts^16^.

Roadless and low-traffic areas could contribute to existing protected areas as has been observed in Europe^16^ and the United States^22^. Some countries already recognise the ecological importance of roadless areas, such as Germany’s Unfragmented areas by traffic (UAT) or the USA’s Inventoried Roadless Areas^18^, with the USA even extending legal enforcement to include roadless areas under the Roadless Conservation Rule of 2001 and the Wilderness Act of 1964. In 2022, the Greek government was the first EU Nation to introduce policy that prevented road construction in six Natura 2000 mountain regions and, using detailed mapping of roadless areas, identified locations for protection under a national roadless policy^23^. A European Roadless Rule has also been proposed that would protect at least 2% of land as road-free areas^24^, although there has been no formal uptake of that in Great Britain (GB). In parts of GB, The Nature Recovery Network is working to connect habitats^25,26^, and there is clearly momentum to include roadless areas within wider protected networks, but for optimum value we need to know where these areas occur and their habitat type. On a global level only 9.3% of roadless areas meet the IUCN criteria of being protected^1,27^ meaning that 90% of these important ecological patches in the landscape could still be subject to other anthropogenic stressors (for example, hunting). To achieve ambitious protected area goals, such as the Global Biodiversity Framework’s “30 by 30” target to protect 30% of land by 2030^28^, efforts need to focus not only on improving existing protected areas for wildlife but also to increase their size and connectivity with other areas.

Roads form a network across countries, intersecting and breaking up habitats into smaller, fragmented landscape patches^29^. This fragmentation can restrict or bisect animal home ranges; species with larger home ranges are more likely to encounter road-effect zones and/or cross roads more frequently than those with smaller home ranges, thus potentially putting them at greater risk of WVCs and increasing exposure to road-related stressors^30,31^. The reduction in ecological connectivity because of fragmentation can also therefore have knock-on effects for population viability and species conservation^32,33^, making it a key threat faced by wildlife in the Anthropocene^34^. Therefore, identifying and quantifying roadless areas is important because they hold inherent ecological value as refuges that remain largely unaffected by the effects of road^16^. Yet, it is estimated that 12% of land in GB is roadless (more than 1 km from a road;^35^), compared with 80% globally^1^.

Quantifying the location, extent, habitat type and level of protection of roadless areas is important for identifying areas of ecological value and strategically expanding conservation efforts. Additionally, the availability of roadless areas of a given size gives insight into how many are large enough to support the home range and movement of given species, ensuring their population viability. Once this is known, the information could potentially be used to expand existing protected areas and subsequently reduce fragmentation^16,17^. Selva et al.^16^, for example, propose that roadless and low-traffic areas in Europe should be a focus for conservation efforts and urgent inventories are needed.

In this study, we characterised the number, location and size of roadless areas in GB using three different road effect zone sizes. Secondly, with the most commonly reported road effect zone size (1 km) in the current literature, we assessed the land cover types associated with roadless areas, the level of fragmentation, the ecological status of roadless areas and the extent to which roadless areas coincide with protected land. Finally, we compared roadless area size to the home range size of species frequently involved in WVCs, to explore how fragmentation may contribute to WVC-risk for different species.

## Methods

### Roadless areas

Roads were extracted and mapped across Great Britain from OpenStreetMap (OSM), an open-access geographic database, in QGIS downloaded 10th August 2022;^36^. OSM is a volunteer-contributed database which was primarily set up to digitise the world’s roads^37^. The completeness of the road networks within OSM depends upon where volunteers are situated^38^ and, historically, concerns have been highlighted about the coverage of the database decreasing in more rural areas^39^. Despite this, it is now believed that the OSM road map is more than 80% complete across the globe and for the UK, this estimate is as high as 99–100%^37^. Road types included in this study were: motorway, trunk, primary, secondary, tertiary, unclassified, residential and living streets (the smallest type of residential road where priority is given to pedestrians and cyclists over vehicles; also known as ‘Home Zones’), and the corresponding ‘link roads’ where available^40^ (Supplementary Fig. 1). We only included paved roads, and excluded those roads designated as, for example, ‘tracks’, to restrict our analyses to roads regularly frequented by vehicles. See Supplementary Table 1 for total road length for England, Scotland and Wales, and a breakdown of the total length of each road class across GB.

Roadless areas were defined, for the purposes of this study, as patches of land beyond a road effect zone^1^. The road effect zone is commonly taken as 1 km, but because the effects of roads can vary depending on the types of effects (noise, pollution, etc.) and species in question, we applied three different buffer sizes (100 m, 500 m or 1 km) equivalent to a range of biotic and abiotic effects^13–15^. Everything outside these buffer zones was classed as a roadless area, or ‘patch’.

We extracted the size (in km^2^) of all roadless patches for each of the three buffer sizes. A Generalised Linear Model (GLM) with gamma distribution (link = "inverse”) was used in R version 4.3.0^41^ to test whether the average size of roadless patches differed between countries within GB (England, Scotland and Wales), for each of the three road effect zones. In addition, we quantified landscape fragmentation for each road effect zone size by using the FragScape plugin in QGIS to calculate the Cross-Boundary Connections Effective Mesh Size (CBC_MSIZ) for each country and across the whole of GB^42,43^. The effective mesh size measures the likelihood that two randomly chosen points will fall within the same patch, by calculating the average size of roadless patches, weighted by area. The higher the effective mesh size, the less fragmented the landscape. Including cross-boundary connections mitigates the issue that country boundaries may intersect a roadless patch and gives a more accurate representation of fragmentation^43^. As it is the most common road effect zone used for landscape-level analyses^1,17^, only data relating to roadless patches produced using a 1 km-buffer were used in analysis hereafter.

### Habitat types, ecological status and protected area coverage

The habitat type associated with roadless patches was investigated by overlaying UKCEH Land Cover 2021 land parcels^44^ with all 1 km-buffer roadless patches in QGIS. The 21 different land cover types included: arable, bog, fen, freshwater, grassland (acid, calcareous, neutral, improved and heather), heather, rock (inland, littoral and supralittoral), saltmarsh, saltwater, sediment (littoral and supralittoral), suburban, woodland (coniferous and deciduous) and urban. We cropped the land cover parcels to the roadless patch boundaries and extracted the total percentage cover of each land cover type across all roadless areas in each country, and GB-wide. To quantify the ecological value of roadless patches we extracted the mean ecological status score for each patch by overlaying the UK ecological status map version 2^45^. The ecological status map represents species richness for 11 broad taxonomic groups across the UK at a 10 km^2^ scale; see Dyer et al. (2016) for detailed methods on how status is calculated. Grid squares that overlapped, intersected, were within or contained a roadless patch were used to calculate the mean ecological status of each patch, including for patches smaller than 10 km^2^.

To assess the protected area coverage of roadless areas, boundaries and designations were downloaded from www.protectedplanet.net^46^ (downloaded 23rd March 2023), as per Starnes et al.^47^. Only protected areas with an IUCN classification were included in the study, excluding those where an IUCN designation was ‘not reported or assigned’. Protected area categories, with increasing levels of protection, were as follows: Protected landscape/seascape; Protected area with sustainable use of natural resources; Habitat/species management area; Natural monument or feature; National park; Strict nature reserve. For more information on the definitions of IUCN designations, see^48^. Where protected area designations overlapped, the designation with the greatest level of protection was retained by using the ‘Difference (multiple layers)’ tool in QGIS. Protected areas were then separated by their IUCN designation and summarised by size. Overlap analysis was carried out in QGIS between roadless and all protected areas, and the percentage overlap calculated, both overall and for each of the IUCN designations. The total area of roadless patches that did not fall within protected areas was calculated. In addition, we carried out a GLM to assess the relationship between mean ecological status of roadless patches with percentage protection and the size (area) of the roadless patch. A gaussian family was used with an ‘identity’ link function, and the natural log of roadless patch size was used to improve model fit. There was no significant interaction between the size of roadless patches and the percentage protected so the interaction was not included in the final model.

### WVC-risk and roadless patch size

Each roadless area is a patch of land bounded by roads, and the greater the number of patches within a given area of landscape, the greater the degree of fragmentation. To gain an overview of how fragmentation might affect WVC-risk, we investigated the proportion of patches large enough to support a given species’ estimated home range. We extracted a range of home range estimates from existing literature (see Supplementary Table 2 for values and sources) for the top 10 most reported mammal species to a UK-wide citizen science roadkill-recording project (The Road Lab: www.theroadlab.co.uk) (e.g.^6,49^). Where possible, home range estimates were taken from studies within the UK. If these were not available, home range estimates from outside of the UK were also included. The mean and standard error of home range estimates were calculated for each species, along with the overall mean across species (see Supplementary Table 2 for values and sources). These species included: European badger *Meles meles*, red fox *Vulpes vulpes*, European hedgehog *Erinaceus europaeus*, European rabbit *Oryctolagus cuniculus*, grey squirrel *Sciurus carolinensis*, Eurasian otter *Lutra lutra*, roe deer *Capreolus capreolus*, Reeves’ muntjac deer *Muntiacus reevesi*, European hare *Lepus europaeus* and European polecat *Mustela putorius*. We acknowledge that we have included both native and invasive non-native species in this study. We chose to focus on those species most frequently recorded as roadkill because of our interest in roadless areas in the context of WVCs, rather than focusing on the species’ ecological value within the UK. The proportion of roadless areas that were large enough to encompass the mean home range of each species was calculated, and for one standard error above and below the mean home range. We only included those roadless areas that fell within the extent of live occurrences for each species within GB (to limit it to only those roadless areas that are ‘available’ to each species), with current known live species distributions being downloaded from the National Biodiversity Network (NBN) from between 2014–2023. Only ‘accepted’ or ‘accepted – considered correct’ data were included. Distribution data were plotted in QGIS and a concave alpha hull polygon was applied for each species separately (with a threshold of 0.05; Supplementary Fig. 2). This polygon represented each species’ current extent and only roadless areas that fell within, intersected or touched the polygon were included.

## Results

### Roadless areas

**Fig. 1:**
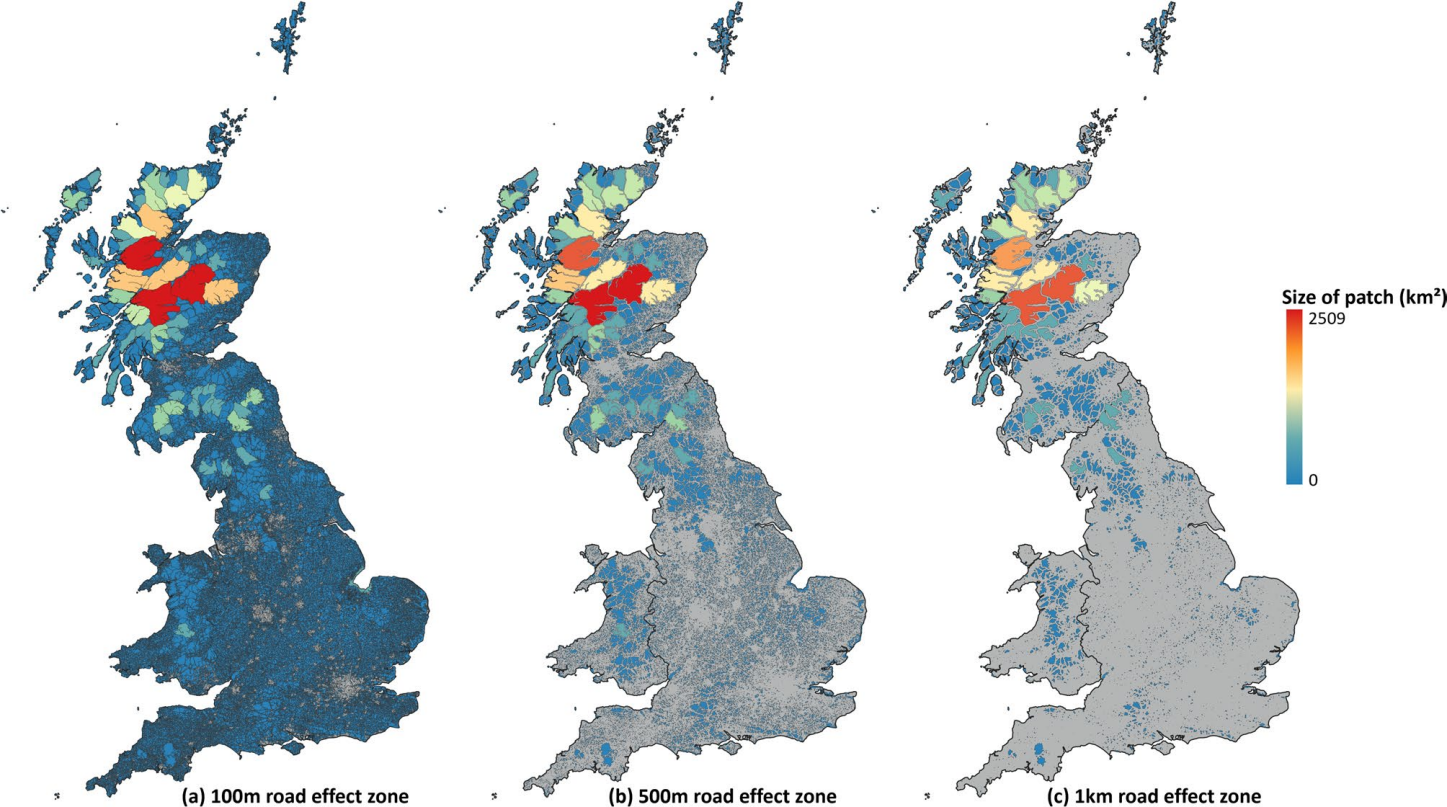
Roadless areas across Great Britain. Using three different road effect zones: (**a**) 100 m, (**b**) 500 m and (**c**) 1 km. The size (area in km^2^) of each patch is also shown by colour, with larger roadless areas being depicted by more red colours and smaller by more blue. (**c**) is publicly accessible on QGIS cloud: https://qgiscloud.com/SarahRaymond/Roadless_areas_GB/.

The total number of roadless patches across GB increased with decreasing road effect zone from 6,138 (1 km road effect zone) to 29,164 (500 m road effect zone) to 93,561 (100 m road effect zone) (Table 1). The mean size of roadless patches ranged from 1.89 km^2^ ± 0.07 (SE) (100 m road effect zone), to 2.85 km^2^ ± 0.002 (500 m road effect zone) and to 7.77 km2 ± 0.85 (SE) (1 km road effect zone) (Table 1). The majority (71–74%) of roadless patches were smaller than 1 km^2^ in size and a small minority (0.002–0.014%) were greater than 100 km^2^ (Fig. 1; Supplementary Fig. 4). This highly over-dispersed pattern of many small patches and few large ones is reflected in a very high variance-to-mean ratio of 249.3 (100 m road effect zone), 420.1 (500 m road effect zone) and 564.4 (1 km road effect zone). Considering the different road effect zones, roadless areas in GB constitute 20.6 to 76.6% of all terrestrial land (20.6% - 1 km road effect zone; 36% - 500 m road effect zone; 76.6% - 100 m road effect zone; Table 1).

**Table 1:** A summary of roadless areas produced using different road effect zones (100 m, 500 m and 1 km), including a breakdown of the number, total area and mean and median roadless area across England, Scotland and Wales, which have a total land area of 130,824 km^2^, 79,364 km^2^ and 20,959 km^2^, respectively. Cross-boundary connections effective mesh size (here denoted as ‘Mesh size – fragmentation’), a measure of fragmentation, is shown, with lower numbers indicating higher levels of fragmentation.

Road effect zone	Country	Count of roadless patches	Total extent of roadless areas (km^2^)	Roadless patch size range (km^2^)	Mean roadless patch size (km^2^)	Median roadless patch size (km^2^)	% of land that is roadless	Mesh size–fragmentation (km^2^)
100 m	England	73,483	90,802	2.95 × 10^–12^–638.9	1.24	0.161	69.4	20.4
	Scotland	11,561	69,977	1.15 × 10^–11^–2,509.3	6.05	0.313	88.2	516.6
	Wales	8,517	16,226	2.72 × 10^–11^–399.6	1.91	0.298	77.4	37
	GB	93,561	177,005	2.95 × 10^–12^–2,509.3	1.89	0.183	76.6	192.3
500 m	England	20,838	27,374	7.5 × 10^–10^–530.2	1.31	0.236	20.9	10.7
	Scotland	5,309	49,467	9.84 × 10^–10^–2,323.7	9.32	0.385	62.3	420.3
	Wales	3,017	6,260	2.01 × 10^–9^–310.9	2.07	0.212	29.9	18.5
	GB	29,164	83,101	7.5 × 10^–10^–2,323.7	2.85	0.253	36	152.1
1 km	England	3,691	8,106	1.14 × 10^–8^–419.7	2.2	0.185	6.2	5.8
	Scotland	1,882	36,889	3.85 × 10^–8^–2,124.3	19.6	0.636	46.5	332.2
	Wales	565	2,723	5.51 × 10^–9^—224.9	4.82	0.211	13	8.1
	GB	6,138	47,718	5.51 × 10^–9^–2,124.3	7.77	0.258	20.6	118.1

Roadless patches differed significantly in size between countries for all road effect zones (e.g. Supplementary Fig. 3). Roadless patches were, on average, significantly larger in Scotland than England and Wales for all road effect zones, and significantly larger in Wales than in England for all but the 500 m road effect zone, where there was a non-significant difference between the two countries (Table 2). This was corroborated by fragmentation which was lowest in Scotland and higher in England (Table 2). Scotland had the greatest percentage roadless area across all road effect zones (88.2%, 62.3% and 46.5% respectively), and larger individual patches on average than either England or Wales (Table 1; Fig. 1). England had the largest number of individual patches, with more than half of patches situated in England (60.1–78.5%), but the lowest overall percentage of land that is roadless (Table 1). Although a much smaller country than either England or Scotland, Wales was intermediate with respect to the percentage roadless area available, as well as (by most metrics) the average patch size (Table 1). To support conservation planning, we provide an open access searchable map of the location, size, protected status and ecological status of roadless areas across GB (available at: https://qgiscloud.com/SarahRaymond/Roadless_areas_GB/).

**Table 2:** Results of the generalised linear models into the size of roadless areas between countries. The size of roadless areas (in km^2^) differed significantly by country (England, Scotland and Wales) across all three road effect zone sizes (100 m, 500 m and 1 km).

Road effect zone	F-statistic	Degrees of freedom	Null deviance	Residual deviance	R^2^-value	AIC	*P*-value
100 m	537	2	584,383	548,794	0.061	33,591	< 0.0001
500 m	238	2	170,159	148,556	0.127	40,224	< 0.0001
1 km	82	2	46,453	40,062	0.138	12,242	< 0.0001

### Habitat types and protected area coverage

**Fig. 2:**
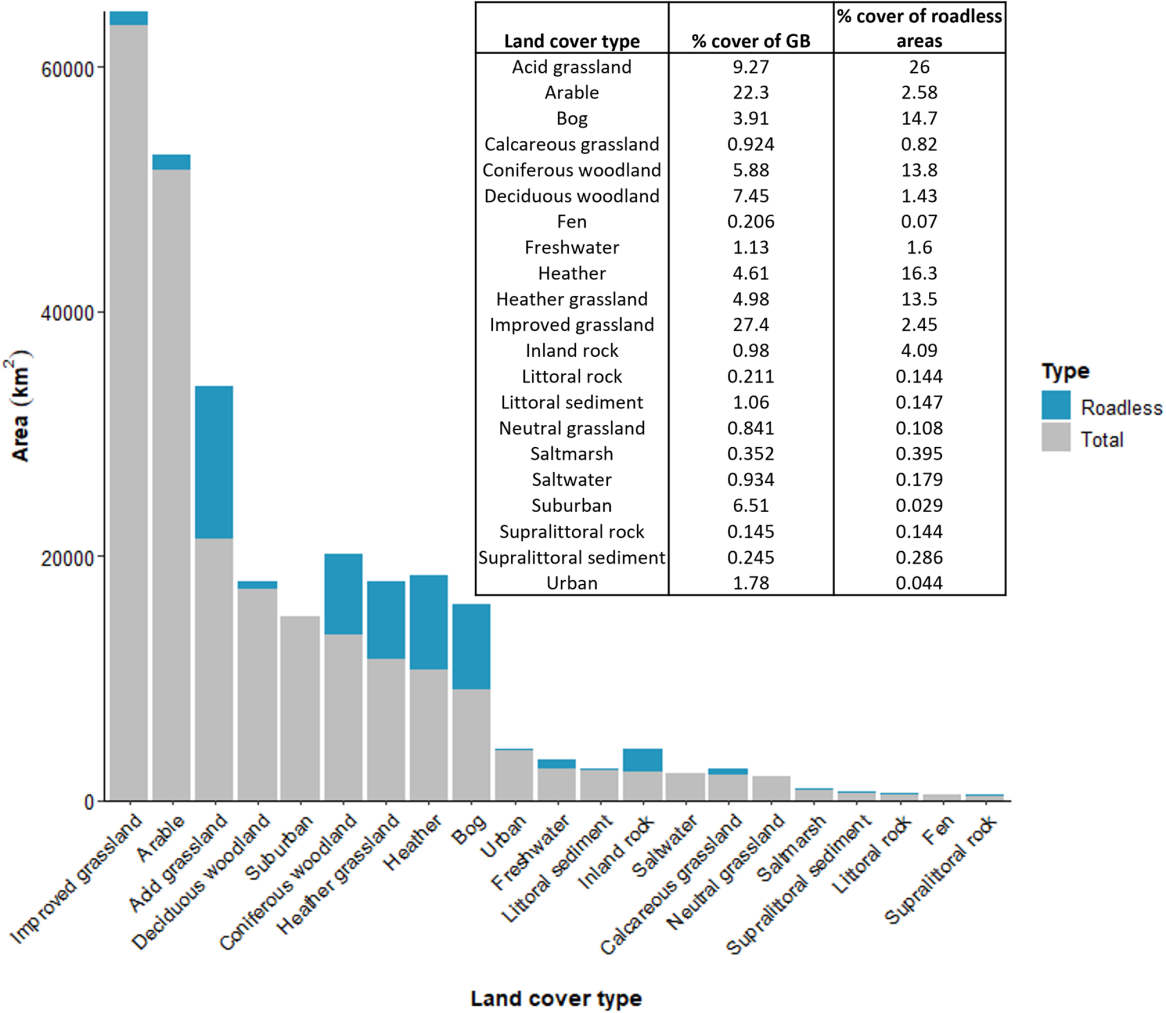
Land cover of roadless areas. The total area (in km2) that each land cover type (from UKCEH Land Cover 2021 land parcels) contributes to roadless areas (specifically, those produced using the 1 km road effect zone; shown in blue) compared to the total coverage of each land cover type across the whole of Great Britain (shown in grey). The percentage cover of each land cover type within GB and across all roadless areas are shown in the inset table.

The most common land cover type for roadless areas (1 km road effect zone) was acid grassland, covering 12,416 km^2^, followed by heather (7,768 km^2^), bog (7,036 km^2^), coniferous woodland (6,601 km^2^) and heather grassland (6,447 km^2^; Fig. 2). Heather grassland was the second most common land cover for roadless areas in Scotland, whereas in England and Wales, arable and coniferous woodland were the second most numerous land cover types, respectively (Supplementary Table 3). The dominant land cover types in roadless areas did not correspond to the most dominant land cover types overall. For example, improved grassland is the most common habitat across GB but only covered 2.45% of all roadless areas (Fig. 2). Additionally, suburban land cover makes up 6.51% of the area of GB but as little as 0.029% of roadless areas. To see the spatial distribution of different land cover types across the roadless areas, see Supplementary Fig. 5.

### Protected area coverage and ecological status of roadless patches

**Fig. 3:**
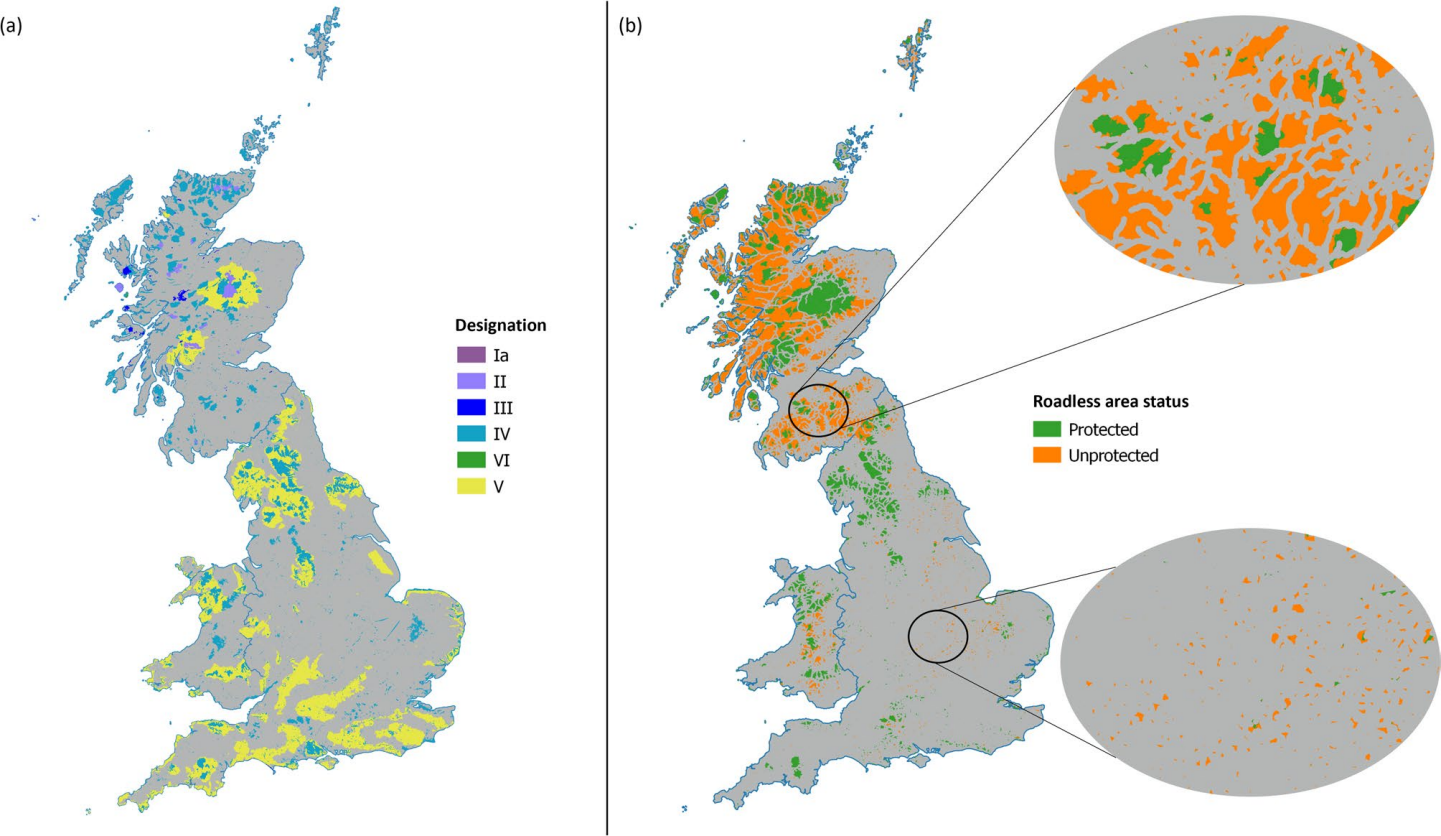
Roadless and protected areas. (**a**) Protected areas fall under six IUCN designations in the UK, indicated here by colour. The categories have decreasing levels of protection in the following order: Ia—Strict nature reserves; II – National park; III – Natural monument or feature; IV – Habitat/species management area; VI – Protected area with sustainable use of natural resources; V – Protected landscape/seascape. In (**b**), roadless patches are separated by colour into those that coincide with protected areas (green), and those that are not protected (orange). Two sections are magnified to show how protected areas may only overlap small sections of each roadless patch, or none at all. A searchable map of Fig. 3b is available at: https://qgiscloud.com/SarahRaymond/Roadless_areas_GB/.

Protected areas covered 62,601 km^2^ of the terrestrial surface of GB (Fig. 3a; Supplementary Table 4), equating to approximately 27% of the terrestrial land mass. Overall, 47% of roadless areas were protected (Fig. 3), although frequently roadless patches were only partially covered by protection rather than the whole patch (Fig. 3b). Habitat/species management areas covered the largest area (29.3%, 13,980 km^2^), but only 0.003% of all roadless areas were classified as strict nature reserves, with the greatest protection (Supplementary Table 4). A further 53% of potentially high ecological value, but currently unprotected roadless areas, could therefore be incorporated into a wider protected area network (Fig. 3b), covering a total of 25,524 km^2^, the equivalent of approximately 11% of GB terrestrial land. There was generally a positive linear trend between roadless area patch size and the protected area within each patch (Supplementary Fig. 6). The magnified areas in Fig. 3b demonstrate how some roadless areas only have a portion of their extent protected, and how protected areas are fragmented by roads.

**Fig. 4:**
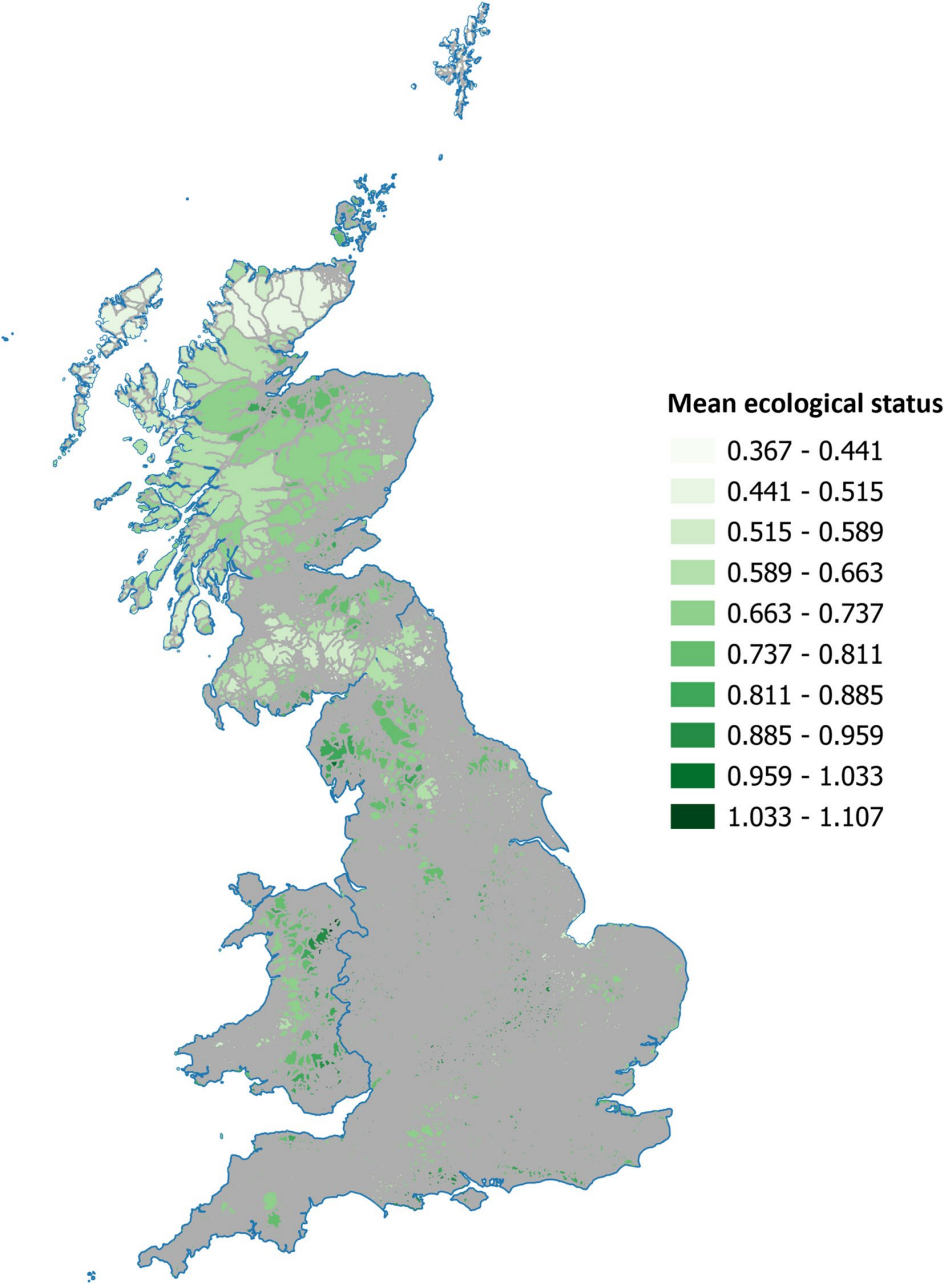
Mean ecological status of roadless areas. The mean ecological status of each roadless patch as estimated from overlapping the UK Ecological Status Map version 2^45^ with each patch. Where multiple 10 km^2^ grid squares from the ecological status map overlapped with a roadless patch, the mean ecological status value was calculated for that patch. Lighter colours indicate lower mean ecological status, and darker colours indicate higher mean ecological status. A searchable map of Fig. 4 is available at: https://qgiscloud.com/SarahRaymond/Roadless_areas_GB/.

The mean ecological status of roadless patches varied between 0.367 and 1.107, with higher values equating to higher levels of ecological status (Fig. 4). There was a weak but significant negative relationship between (logged) area and mean ecological status of roadless patches (F(1, 6051) = 50.8, p < 0.0001, deviance explained = 0.81%), and this relationship was reversed when roadless patches had a level of formal protection, with a weak but significant positive relationship between the percentage of each roadless patch protected and mean ecological status (F(1, 6056) = 180.7, p < 0.0001, deviance explained = 2.87%).

### Roadless area size and WVC-risk

**Fig. 5:**
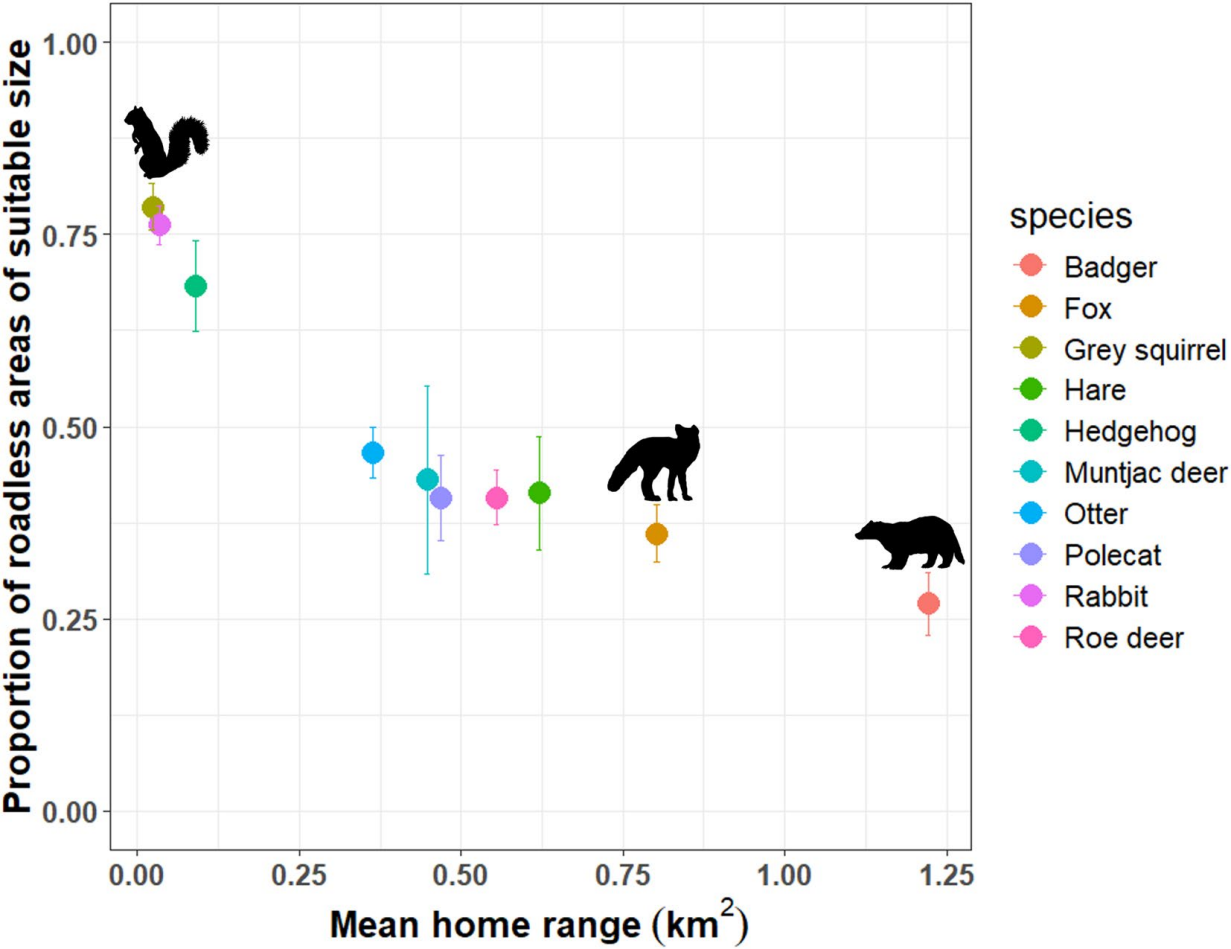
Roadless areas and species’ home ranges. The relationship between the proportion of roadless patches large enough to encompass the individual mean home range of 10 species most commonly reported as wildlife-vehicle collisions (shown in colour). Error bars show the proportion of roadless patches of suitable size for a home range of one standard error above or below the mean. Only those roadless areas that were within the current extent of each species’ live occurrence (based on NBN data), and therefore available to the species, were included (see Supplementary Fig. 2 for an example of how this was carried out). Grey squirrels had the smallest home range and therefore highest proportion of roadless patches of suitable size, whilst badger and fox had the largest home range and lowest proportion of suitable roadless patches.

The mean home range estimate across the road-vulnerable mammal species included in this study was 0.46 km^2^ (± 0.12), with the smallest mean home range being attributed to grey squirrels (0.02 km^2^), and the largest to badgers (1.22 km^2^) (Fig. 5). Within their current known distribution, less than half of all roadless patches were large enough to support mammals with larger mean home ranges, including: badger, fox, hare, otter, polecat, roe deer (Fig. 5), meaning we could expect these species to be most vulnerable to WVC-risk because they will need to cross roads frequently in order to meet the ecological requirements associated with their given mean home range. For example, only 27% and 36.1% of roadless patches were equal to or greater than the mean home range of a badger or fox, respectively (Fig. 5). In general, mammals with larger home ranges had far fewer roadless patches large enough for their average home range than those with smaller home ranges (Fig. 5). Species with smaller home ranges, like the grey squirrel, rabbit and hedgehog, had a greater proportion of roadless areas of a suitable size within their current extent (78.6%, 76.2% and 68.3%, respectively; Fig. 5).

## Discussion

In this study, we evidence the extensive fragmentation caused by roads within GB. Over 70% of roadless patches were smaller than 1 km^2^ in size, with less than half of all roadless patches large enough to encompass the home range of common British wildlife species. This pattern highlights the pressure that linear infrastructure can impose on wildlife, particularly mammals with large home ranges that exceed the size of most roadless patches. Roadless areas have been proposed as conservation targets for functional ecosystems, but our work highlights that 53% of all roadless areas were not covered by existing protected habitat designation. There is a need to increase protection of roadless areas and connectivity between them—but the largest roadless patches are predominantly acid grassland, which tends to be heavily grazed, and have low ecological status scores. However, the ‘value’ of roadless areas goes beyond quantified ecological status, and large areas especially may play important roles in terms of ecosystem services, such as carbon storage, sequestration and flood management^20,21^.

### What are the features of roadless areas in Great Britain?

Roadless areas in GB, on average, constituted patches of land that were substantially smaller than the average across Europe (only 7.77 km^2^ compared to 48 km^2^, from a 1 km road effect zone)^1^. In England, which has the highest human population density, the mean size of roadless patches was just 2.2 km^2^. The largest roadless patches were found in Scotland, coinciding with the Scottish Highlands and Cairngorms National Park, and likely exhibited relatively low road density due to higher levels of elevation and low human population density in these regions^50^. The effects of roads in GB extend over 23.4%, 64% or 79.4% of terrestrial land for 100 m, 500 m and 1 km road effect zones respectively. Whilst a given small patch may be of ecological value due to supporting taxa of conservation importance^50,51^, small, isolated patches (indicative of high fragmentation) typically experience low species persistence, fundamental changes in community compositions and degradation of ecosystem functioning^52^.

Cumulatively, roadless areas in GB were most commonly comprised of anthropogenically managed habitat. Acid grassland, a semi-natural habitat frequently managed by livestock grazing^53,54^, was the most common land cover type in both mountainous regions in Scotland, and within larger patches in northern England and central Wales (Supplementary Fig. 5). The second most common land cover type of roadless areas was heather (Fig. 2)—much of this habitat type is found in high elevation regions in Scotland, coinciding with some of the largest roadless areas (Supplementary Fig. 5). Acid grassland and heathland can support a diversity of invertebrates, plants and unique bird species^55,56^; however, these habitats are generally less suitable for mammal species, such as those included in this study and at the greatest risk of WVCs. Roadless areas in GB, therefore, typically consist primarily of small patches of land with relatively high levels of anthropogenic management, and larger patches exist only in those areas of higher elevation.

The ecological ‘value’ of roadless patches is hard to interpret. While we find that roadless patches that are protected are associated with marginally better ecological value (using the UK Ecological Status map version 2^45^) than unprotected patches, the variance explained by the model was very low. While the UK Ecological Status metric provides a convenient, standardised way to compare habitat value it should be used as a broad comparator^57^. Qualitatively, we find acid grasslands, heathlands, bogs, coniferous woodlands, and heather–grassland mosaics dominate in large roadless patches. While the Ecological Status score may be low for some of these habitat types, this does not necessarily reflect the ‘value’ of these habitats in terms of species rarity, endemism or the capacity of these habitats to provide ecosystem services. Bogs and peatlands, for example, are dominated by *Sphagnum* mosses and accumulate peat over millennia, making them significant long-term carbon stores, as well as regulators of water flow, reducing downstream flood risk^58,59^.

### Which species are most at risk due to fragmentation?

The results of this study evidence that wildlife in GB is frequently exposed to the effects of roads, and this is exacerbated for species with a larger home range. The European badger has a mean home range (1.22 km^2^) greater than the majority (73%) of roadless patches. High levels of fragmentation put species with large home ranges under particularly high pressure – they *have* to cross roads for dispersal and resource access and as a result either must adapt and learn how to avoid WVCs, or risk mortality on roads^60^. Indeed, previous research has found that landscape connectivity may be more important than habitat suitability for predicting WVC-risk^61^. Importantly, species with smaller home ranges (such as the European hedgehog and rabbit; Fig. 5) frequently also must cross roads, avoid WVCs and encounter road effect zones^3,5,62^, despite their more localised movements and a greater frequency of roadless patches that encompass their home range.

Understanding species-specific behaviours and how these vary is also important when considering the utilisation of roadless areas for conservation. Using mean home range provides a useful starting point but, for example, home ranges may vary in shape, with sex, and between seasons. For example, some species such as the Eurasian otter and European hedgehog have a relatively small mean home range in square kilometres (only 0.364 km^2^ and 0.09 km^2^, respectively); however, these species may have more linear and longer ranging movements, and therefore more frequently encounter roads, than their home range suggests. Otters can have a linear home range of 5–40 km along a river^63–65^, and hedgehogs have been recorded travelling close to 2 km in a single night^66,67^. Interactions with roads also depend on whether an individual’s range is near the edge of a roadless patch or in the middle, which is why edge effects of fragmentation are important^68,69^; the smaller the patch, the greater the proportion of individuals that will have a home range bordering a road effect zone. Additionally, differences in home range can occur between sexes of the same species, with male home ranges frequently being larger than females (e.g.^70,71^), which could lead to a disproportionate effect of fragmentation on male individuals. The effects of fragmentation could also vary with seasons, as has been demonstrated for wildlife-vehicle collision risk, with high risk largely coinciding with species-specific breeding and mating seasons, when species are most active^49^. Finally, differences in the effects of fragmentation could also occur between populations of the same species in urban versus rural environments. Home ranges across multiple taxa tend to be smaller in urban compared to natural environments for both birds and mammals, suggesting that species may adapt their home range in more built-up environments^72^. Regardless of the species and individual variation in home range size, it is clear the high level of fragmentation and the small mean size of roadless patches presents a challenge to wildlife conservation in GB.

### Could roadless areas be incorporated into protected area networks?

Although this study primarily highlights the extent to which the landscape is fragmented, we also propose that linking these areas would not only defragment the landscape but could expand the current protected area networks, to increase overall connectivity of the landscape for wildlife. Whilst the ‘single large or several small’ (SLOSS) debate has existed since the 1970s^73^, recent research has emphasised that combinations of large and small protected areas are optimal for maximising biodiversity^74–76^. Larger protected areas are particularly important for reducing edge effects and for accommodating species with larger home ranges^77,78^. Given that, as previously discussed, most large roadless patches contained potentially less valuable habitat for mammal species and are of lower ecological status, smaller patches could be important for providing habitat for those species most threatened by roadkill. The UK government, along with many other countries, has committed to protect 30% of land by 2030^79,80^. Currently, 27% of British land has some level of protected designation (Fig. 3a); however, designations that are actually effective in protecting nature are as few as 5%^80^. We found that 47% of roadless areas (9.7% of all GB) were covered by existing protected areas, which is slightly greater than the coverage of Natura 2000 sites across European roadless areas^17^. However, typically only a small section of roadless areas was protected, rather than whole patches (Fig. 3b), but we did also find that these protected areas were of marginally higher ecological status compared to unprotected habitats. A practical first step in conservation could be to extend protection across the entirety of those patches where a portion of the patch is already protected, before moving on to protect other patches. If all remaining, unprotected roadless areas were given protected designations (equating to an area of 25,524 km^2^), this could raise the total land mass covered by protected areas within GB to 38%, exceeding the “30 by 30” target. It is more realistic, however, to suggest that larger roadless patches could be protected and connectivity between smaller patches improved. Importantly, roadless areas are unlikely to be completely free of anthropogenic impacts on wildlife and could represent areas of poor ecological value, but defragmentation would certainly help reduce the direct effects of roads, such as wildlife-vehicle collisions^81,82^. Other types of linear infrastructure (such as railways) or human activities that have negative impacts on wildlife may exist within the remaining roadless areas that we have not assessed in this paper. As such, not all roadless areas will be valuable for conservation and a useful next step would be to identify those other sources of anthropogenic pressure that may exist within roadless areas.

Defragmenting and protecting roadless areas could contribute to increasing the overall *extent* of protected areas in GB; however, we need to also prioritise reforming and improving our existing protected areas^80^. Only 44% of existing protected areas in Britain currently meet IUCN criteria for the strictest levels of protection and a large proportion of protected areas are not currently effectively managed or monitored for nature^47^. Additionally, Li et al. (2021) highlights the importance of habitat characteristics when establishing protected areas^83^, rather than focusing solely on increasing the coverage or size of protected areas, particularly as there is a greater cost involved with establishing larger protected areas^78^. Social and political considerations are also hugely important when establishing new, or extending existing, protected areas^84–86^. An important consideration for prioritising which roadless patches to protect is the provision of ecosystem services. As previously discussed, some patches deemed to have ‘low’ ecological status may already be providing important ecosystem services, and there could be social trade-offs between protection and economic growth for stakeholders^87^. The potential impacts of protection on these services should be investigated in the decision-making process. On the other hand, protecting roadless areas could aid in the recovery and restoration of currently degraded ecosystem services^88^, and also play an important role in supporting human health and wellbeing^89,90^. Directed surveys are needed to investigate the ecosystem services currently provided by roadless areas and how protection of roadless areas might affect this.

Our research provides an overview of roadless areas and quantifies fragmentation and ecological value within GB; however, it is not an assessment of how and where patches should be joined. The ‘Biodiversity and Infrastructure Synergies and Opportunities for European Transport Networks’ (BISON) project has produced a European Defragmentation Map by mapping transport infrastructure, habitats, protected areas and existing wildlife crossing structures (overpasses, underpasses and tunnels)^91^. This provides a broad overview of potential areas for defragmentation across mainland Europe and Ireland, but reiterates that actual implementation requires more local scale studies and ground-truthing of data. On the other hand, the Dutch habitat defragmentation program is a good example of where practical measures have actually been taken to improve connectivity at a landscape-wide scale^92^, spending over 410 million euros on defragmentation. The program has used population viability analysis to identify priority defragmentation spots, and by 2018, 126 ecological barriers to movement had been removed, and over 2,100 wildlife crossings had been built^93^. Similar methods could be adopted in GB to explore priority sites for defragmentation. Given that decisions about which patches to link or protect depend on fine scale ecological conditions best assessed through *in situ* surveys, we refrain from recommending specific configurations in this paper. To facilitate practitioner led evaluation, we provide an open access, searchable mapping tool (https://qgiscloud.com/SarahRaymond/Roadless_areas_GB/). Whilst the implementation of wildlife crossings can help reduce WVC occurrence and increase connectivity, to execute this on a large scale would be financially costly^94,95^, so identifying priority areas before installation is key.

## Conclusions

The findings of this study emphasise the extent to which Great Britain is fragmented by roads and the extent of their effects. We show that roads generate novel ecosystems with important implications for wildlife, requiring species to navigate and adapt to a highly fragmented landscape—pressures that are especially acute for wide-ranging species. We highlight the potential for incorporating roadless areas into existing protected area networks, to increase connectivity between patches of land protected for nature and reduce WVCs; however, we also emphasise the need to quantify the value of habitats available to wildlife within protected and unprotected roadless areas. Future research should focus on identifying priority areas for practical interventions, and on improving our understanding of the long-term and future effects of roads on wildlife populations.

## Supplementary Information

Supplementary Information.

## Data Availability

All datasets used in this study are publicly available. Spatial road data can be accessed through the OpenStreetMap plugin in QGIS, and protected area files can be downloaded from www.protectedplanet.net. The UKCEH land cover map can be downloaded from 10.5285/398dd41e-3c08-47f5-811f.-da990007643f. A searchable map of roadless areas can be viewed here: https://qgiscloud.com/SarahRaymond/Roadless_areas_GB/).
