# Supplementary material for: Quantifying roadless areas and fragmentation in the context of wildlife-vehicle collision risk in Great Britain
Source: Sci Rep. 2026 Feb 18;16:3890. doi: 10.1038/s41598-026-36410-8 (PMC12917188; doi:10.1038/s41598-026-36410-8)
Supplement: Supplementary file 1 — Supplementary Information. [file 41598_2026_36410_MOESM1_ESM.docx]

**Supplementary Material**

**Contents**

Supplementary Figure 1: Roads included in this study and their distribution across Great Britain….p.2

Supplementary Table 1: Road lengths by type and country in kilometres……………………………………….p.3

Supplementary Table 2: Home range estimates for species frequently reported as roadkill………..p.4-5

Supplementary Figure 2: Steps involved to subset roadless areas for comparison with species’ home ranges…………………………………………………………………………………………………………………………………………….p.6

Supplementary Figure 3: Roadless patch size by country………………………………………………………………..p.7

Supplementary Figure 4: Roadless patch size by road effect zone…………………………………………………..p.8

Supplementary Table 3: Land cover of roadless areas by country…………………………………………………..p.9

Supplementary Figure 5: The spatial distribution of the 21 different UKCEH Land Cover 2021 types across all roadless areas in Great Britain……………………………………………………………………………………….p.10

Supplementary Table 4: Protected area coverage…………………………………………………………………………p.11

Supplementary Figure 6: Protected area coverage of roadless patches of different size……………….p.12

References……………………………………………………………………………………………………………………………………p.13

**
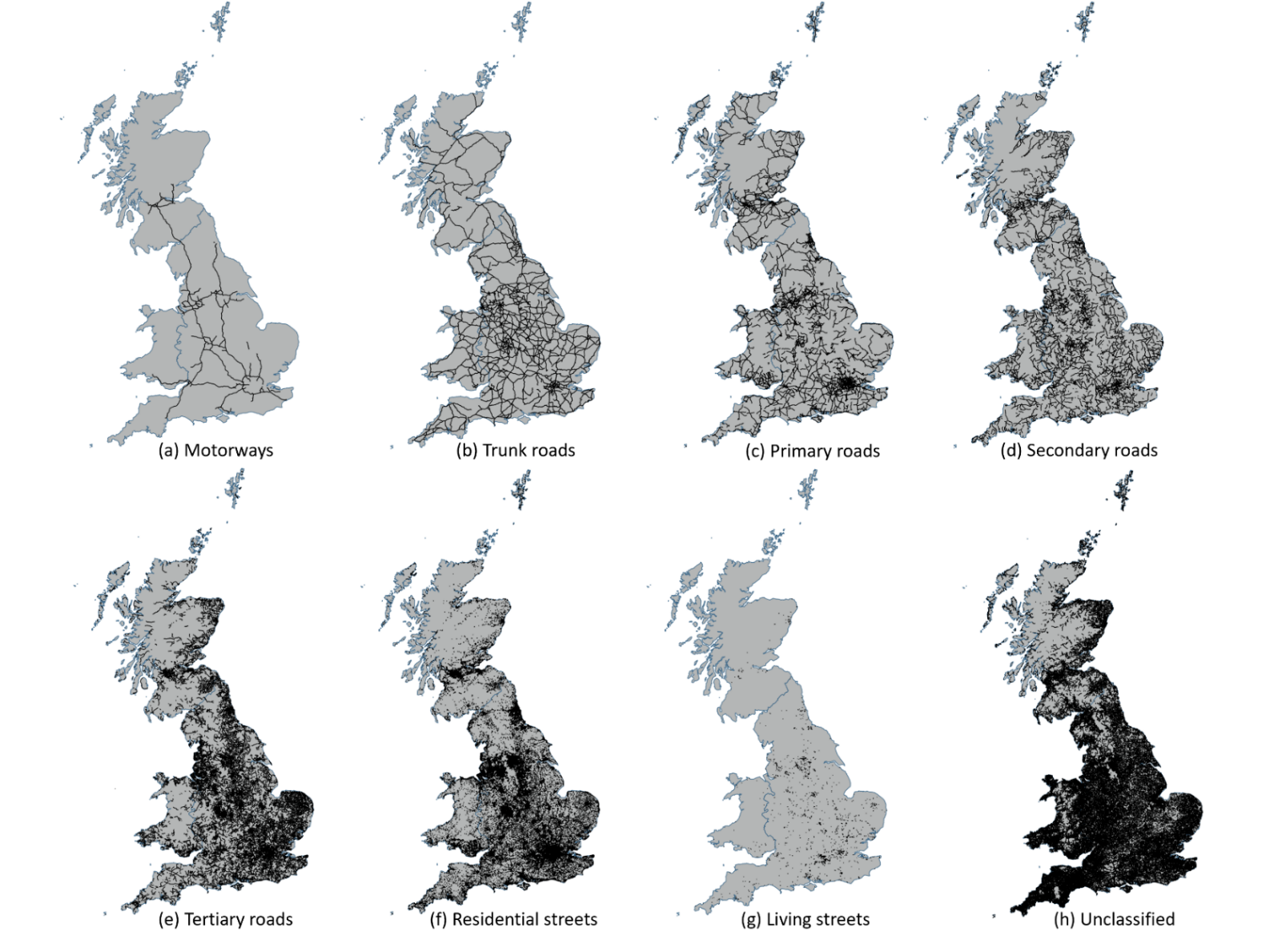
**

Supplementary Figure 1: Roads included in this study and their distribution across Great Britain: (a) motorways, (b) trunk roads, (c) primary roads, (d) secondary roads, (e) tertiary roads, (f) residential streets, (g) living streets and (h) unclassified roads.

Supplementary Table 1: Road lengths by type and country in kilometres. Note that for motorways, trunk, primary, secondary and tertiary roads, road ‘links’ were also included in the length estimate.

| **Country** | **Road type** | **Length (km)** |
| --- | --- | --- |
| **England** | Motorway | 7176.5 |
|  | Trunk | 35188.7 |
|  | Primary | 23329.3 |
|  | Secondary | 26205.1 |
|  | Tertiary | 61724.3 |
|  | Residential | 212945.5 |
|  | Living streets | 657.2 |
|  | Unclassified | 109449.9 |
|  | **Total** | **476676.5** |
| **Scotland** | Motorway | 1094.8 |
|  | Trunk | 3960.5 |
|  | Primary | 8309.9 |
|  | Secondary | 8345 |
|  | Tertiary | 10525.1 |
|  | Residential | 15720.1 |
|  | Living streets | 35.1 |
|  | Unclassified | 18812.1 |
|  | **Total** | **66802.6** |
| **Wales** | Motorway | 318.4 |
|  | Trunk | 5438.8 |
|  | Primary | 4759.1 |
|  | Secondary | 5074.5 |
|  | Tertiary | 5794.3 |
|  | Residential | 9017 |
|  | Living streets | 17.4 |
|  | Unclassified | 25838 |
|  | **Total** | **56257.5** |

**Supplementary Table 2: Home range estimates for species frequently reported as roadkill**. Numerous home range estimates were collected from existing literature for the top 10 mammal species most reported as roadkill to The Road Lab, a UK-wide citizen science roadkill recording project (https://www.theroadlab.co.uk/). The number of estimates for each species is shown as ‘n=’. The mean and standard error were calculated for individual species.

| **Species** | **Range of home range estimates (km^2^)** | **Mean (±SE) home range estimate (km^2^)** | **Sources** |
| --- | --- | --- | --- |
| Eurasian otter *Lutra lutra* | 0.168-0.787 (n=6) | 0.364 (±0.09) | Durbin 1996; Durbin 2006; Ó Néill et al. 2009 |
| European badger *Meles meles* | 0.38-2.18 (n=6) | 1.22 (±0.28) | Gaughran et al. 2018; Kruuk 1978; Kruuk and Parish, 1987; Magowan et al. 2022; Redpath et al. 2023 |
| European hare *Lepus europaeus* | 0.09-1.68 (n=6) | 0.574 (±0.23) | Ferretti et al. 2010; Kunst et al. 2001; Misiorowska 2013; Smith et al. 2004 |
| European hedgehog *Erinaceous europaeus* | 0.007-0.216 (n=5) | 0.09 (±0.04) | Gazzard et al. 2022; Morris 2009; Pettett et al. 2017 |
| European polecat *Mustela putorius* | 0.124-1.23 (n=6) | 0.469 (±0.17) | Birks and Kitchener, 1999; Harrington and Macdonald, 2008 |
| European rabbit *Oryctolagus cuniculus* | 0.013-0.063 (n=5) | 0.034 (±0.01) | Daniels et al. 2003; Hulbert et al. 1996 |
| Grey squirrel *Sciurus carolinensis* | 0.005-0.034 (n=4) | 0.023 (±0.01) | Kenward 1982; Tounzen et al. 2013 |
| Red fox *Vulpes vulpes* | 0.14-2.72 (n=13) | 0.803 (±0.2) | Arnold et al. 2011; Harris 1980; Hofer 1986; Kolb 1984; Newman et al. 2003; Porteus et al. 2024; Reynolds and Tapper 1995; Saunders et al. 1993; Tolhurst et al. 2016; Voigt and Macdonald 1984; White et al. 1996; Woollard and Harris 1990 |
| Reeves’ muntjac deer *Muntiacus reevesi* | 0.108-1.08 (n=3) | 0.448 (±0.31) | Chapman et al. 1993; McCullough et al. 2000 |
| Roe deer *Capreolus capreolus* | 0.202-1.55 (n=9) | 0.555 (±0.14) | Guillet et al. 1996; Morellet et al. 2013; Pandini and Cesaris. 1997; Saïd and Servanty 2005; Rossi et al. 2003; Saïd et al. 2009 |

Supplementary Figure 2: Steps involved to subset roadless areas for comparison with species’ home ranges. An example of the steps involved to extract just those roadless patches within the current range of live species’ occurrences for the 10 most-reported mammal species to The Road Lab. In this example, muntjac deer is used. (a) Live muntjac occurrences were downloaded from NBN from 2014-2023 (brown points), then (b) a concave alpha hull polygon (shown here in light blue) with a 0.05 threshold was created around the data points, and (c) only roadless patches that fell within, intersected or touched the polygon were then used for the comparison of home range versus roadless patch size. Roadless patches that were included are shown in dark blue and those excluded are shown in pink. We retained those roadless patches that intersected or touched the concave hull because this enabled the inclusion of roadless patches at the edge of the species’ range or only partially covered by a species’ range.


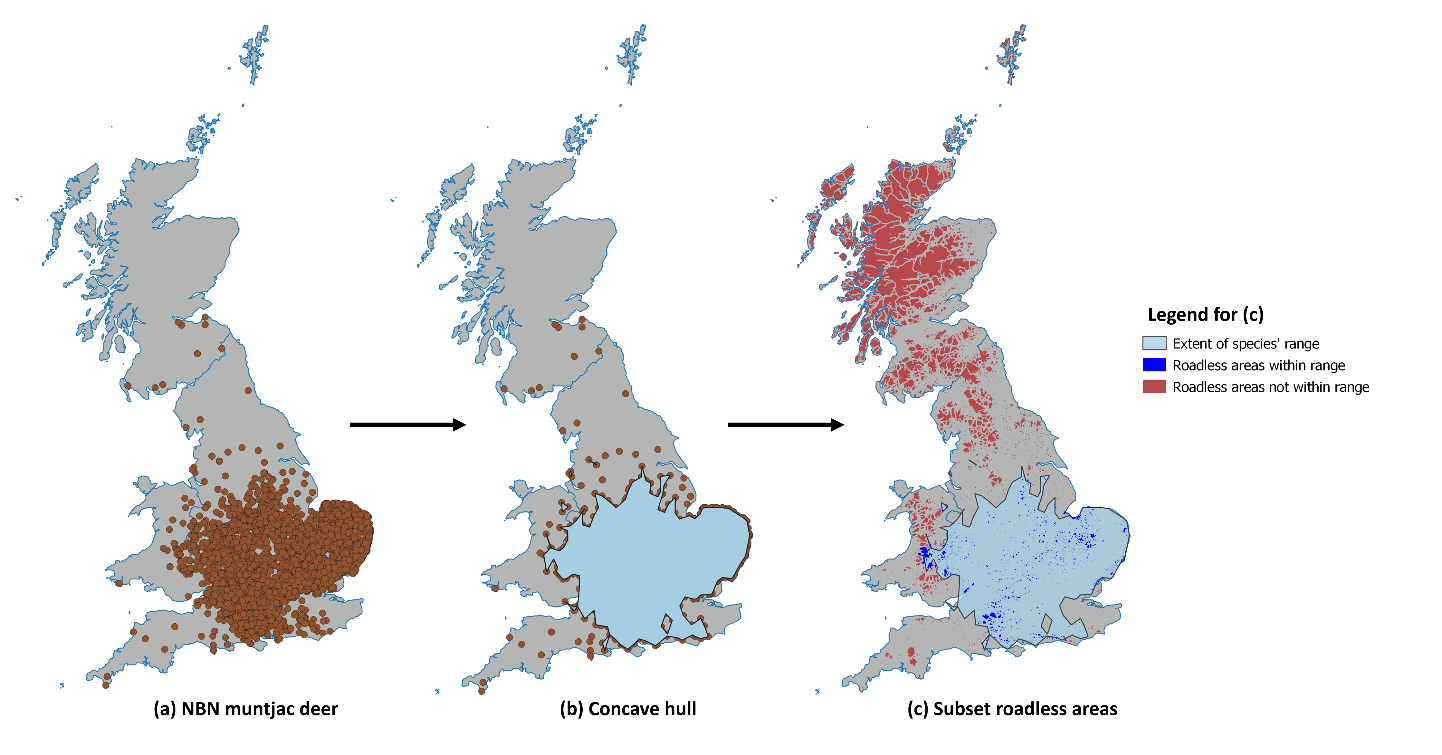

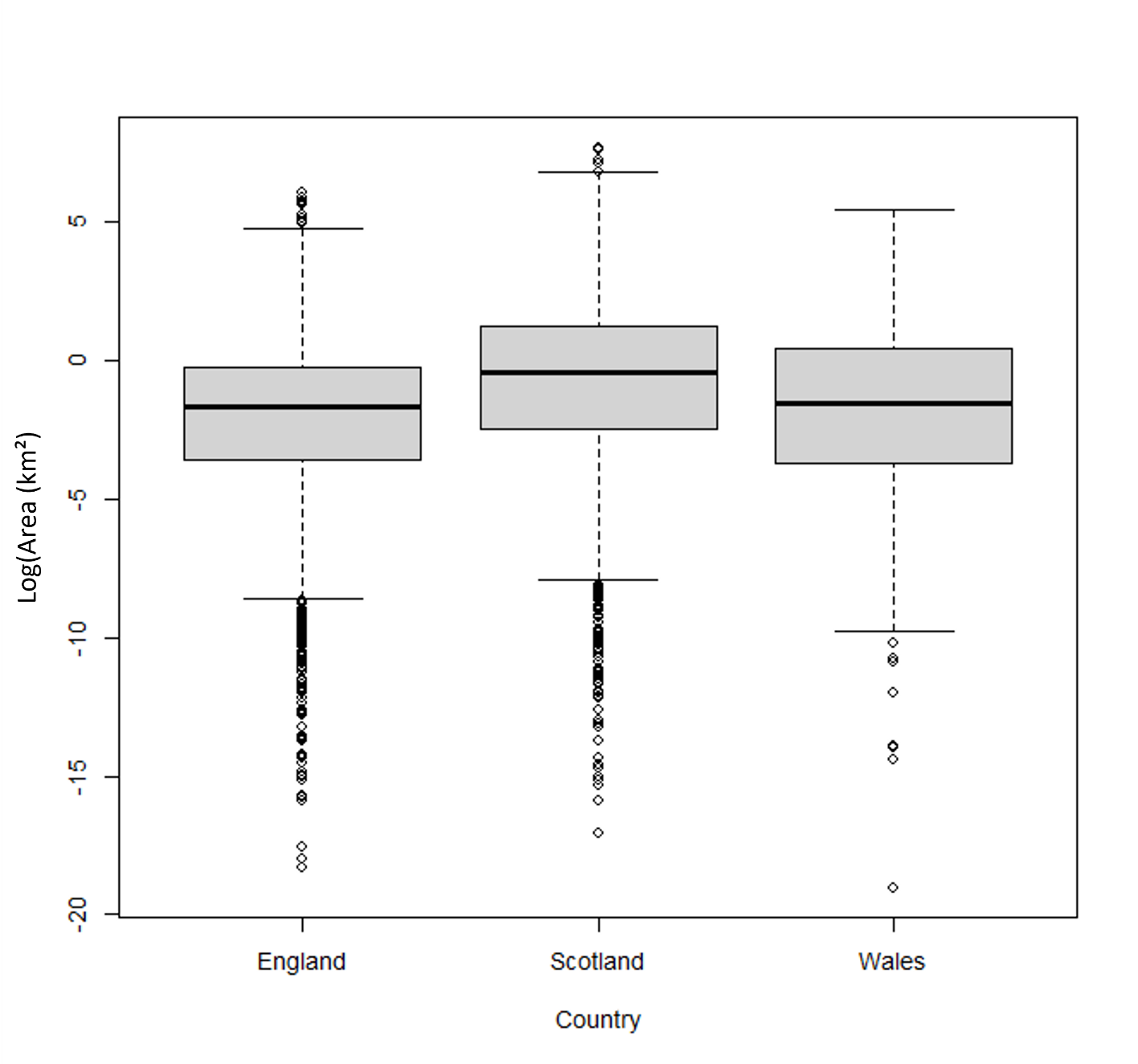


Supplementary Figure 3: Roadless patch size by country. Comparison of roadless patch size (the log of area in km^2^) in England, Scotland and Wales, produced using the 1 km road effect zone.

Supplementary Figure 4: Roadless patch size by road effect zone. Distribution of the logged (base 10) size (area in km²) of roadless patches across Great Britain, produced using the (a) 100 m, (b) 500 m or (c) 1 km road effect zone. Most roadless patches were smaller than 1 km^2^. For reference, zero here represents 1 km^2^ roadless patches, -10 is equal to 1 x 10^-10^ km^2^ and 5 is equal to 100,000 km^2^. .


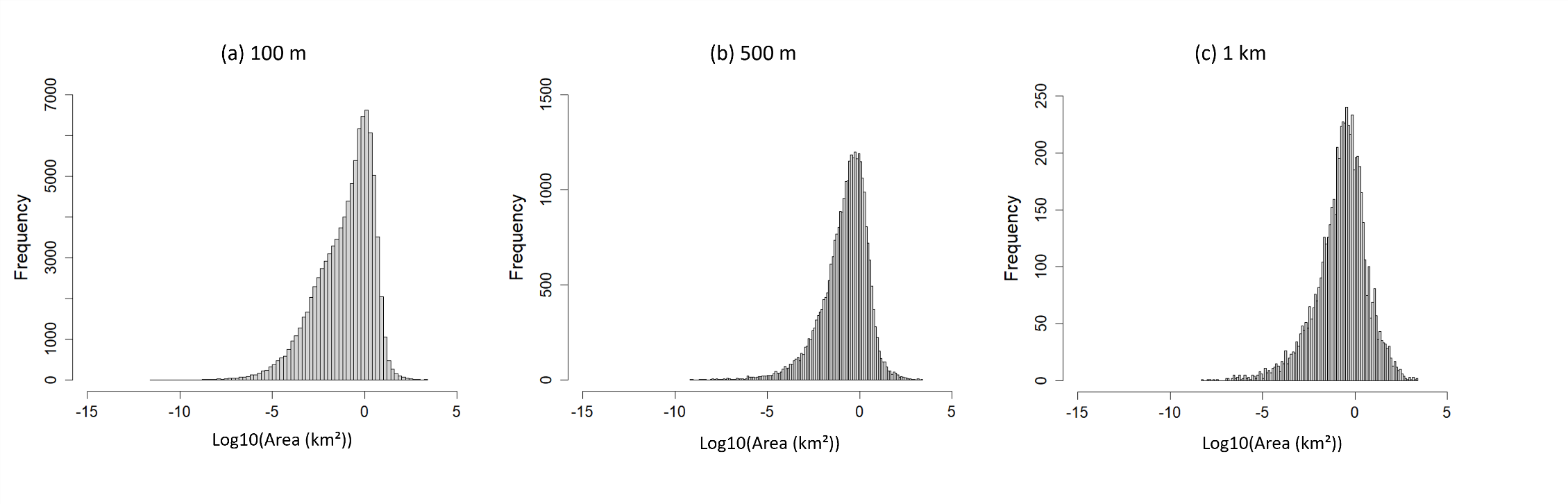


**Supplementary Table 3:** **Land cover of roadless areas by country.** A breakdown of the total area (in km^2^) that each land cover type (from the UKCEH Land Cover Parcels 2021) contributes to all roadless areas across England, Scotland, Wales and Great Britain (total). The top five land cover types for each country and overall are highlighted in grey.

|  | **Area (km^2^)** | | | |
| --- | --- | --- | --- | --- |
| **Land cover type** | **England** | **Scotland** | **Wales** | **Total** |
| Deciduous woodland | 309.8 | 333.3 | 38.6 | 681.7 |
| Coniferous woodland | 867.5 | 5238.1 | 495.3 | 6600.9 |
| Arable | 1140.6 | 83.9 | 6.7 | 1231.1 |
| Improved grassland | 569.1 | 528.8 | 69.0 | 1167.0 |
| Neutral grassland | 36.6 | 3.5 | 11.2 | 51.3 |
| Calcareous grassland | 376.5 | 14.0 | 0.0 | 390.5 |
| Acid grassland | 1772.8 | 9113.9 | 1529.1 | 12415.9 |
| Fen | 28.2 | 2.3 | 1.6 | 32.1 |
| Heather | 832.1 | 6645.5 | 290.0 | 7767.7 |
| Heather grassland | 272.1 | 6128.6 | 46.4 | 6447.2 |
| Bog | 1491.4 | 5397.7 | 146.6 | 7035.7 |
| Inland rock | 21.4 | 1926.0 | 4.8 | 1952.1 |
| Saltwater | 54.4 | 30.7 | 0.5 | 85.6 |
| Freshwater | 45.5 | 701.6 | 14.8 | 761.9 |
| Supralittoral rock | 5.9 | 54.4 | 8.1 | 68.5 |
| Supralittoral sediment | 33.5 | 81.0 | 22.0 | 136.5 |
| Littoral rock | 3.6 | 65.0 | 0.0 | 68.5 |
| Littoral sediment | 47.0 | 18.4 | 4.9 | 70.3 |
| Saltmarsh | 139.2 | 27.1 | 22.3 | 188.6 |
| Urban | 15.2 | 3.2 | 2.5 | 20.9 |
| Suburban | 8.4 | 3.8 | 1.4 | 13.6 |


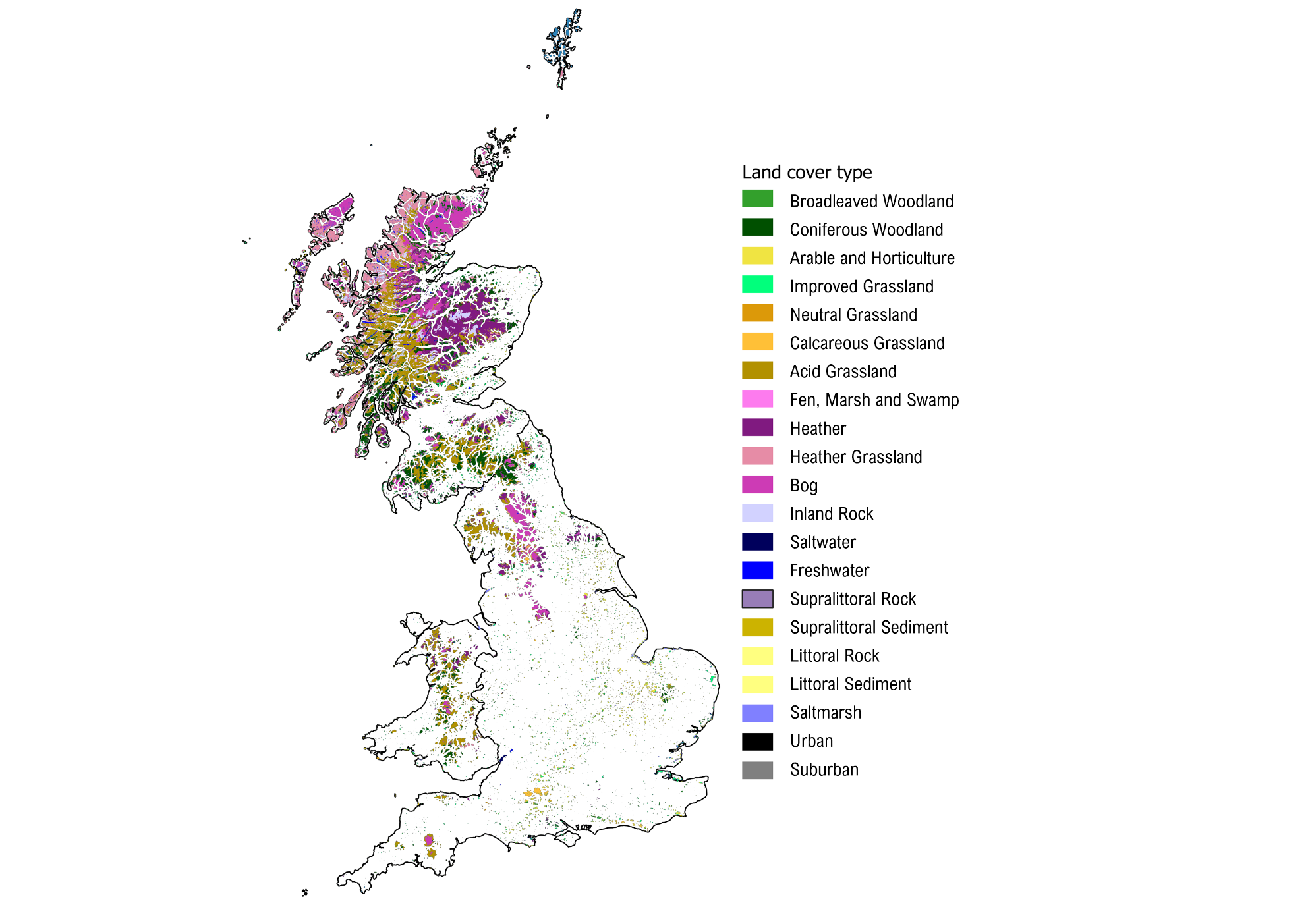


Supplementary Figure 5: The spatial distribution of the 21 different UKCEH Land Cover 2021 types across all roadless areas in Great Britain. The colour corresponds to the different land cover type.

**Supplementary Table 4:** **Protected area coverage**. A breakdown of the total coverage of IUCN protected areas across Great Britain, the area of roadless areas that fall under each category, and the total percentage of roadless areas that this equates to.

| **Protected area category** | **Total coverage in GB (km^2^)** | **Area of overlap with roadless areas (km^2^)** | **Percentage of total roadless area (%)** |
| --- | --- | --- | --- |
| Ia – Strict nature reserve | 7.3 | 1.5 | 0.003 |
| II – National park | 1581.5 | 1321.4 | 2.8 |
| III – Natural monument or feature | 578.9 | 321.5 | 0.7 |
| IV – Habitat/species management area | 25415.1 | 13980.4 | 29.3 |
| VI – Protected area with sustainable use of natural resources | 26.8 | 9 | 0.02 |
| V – Protected landscape/seascape | 34991.8 | 6560.1 | 13.7 |
| Unprotected | 25524 | - | 53.5 |


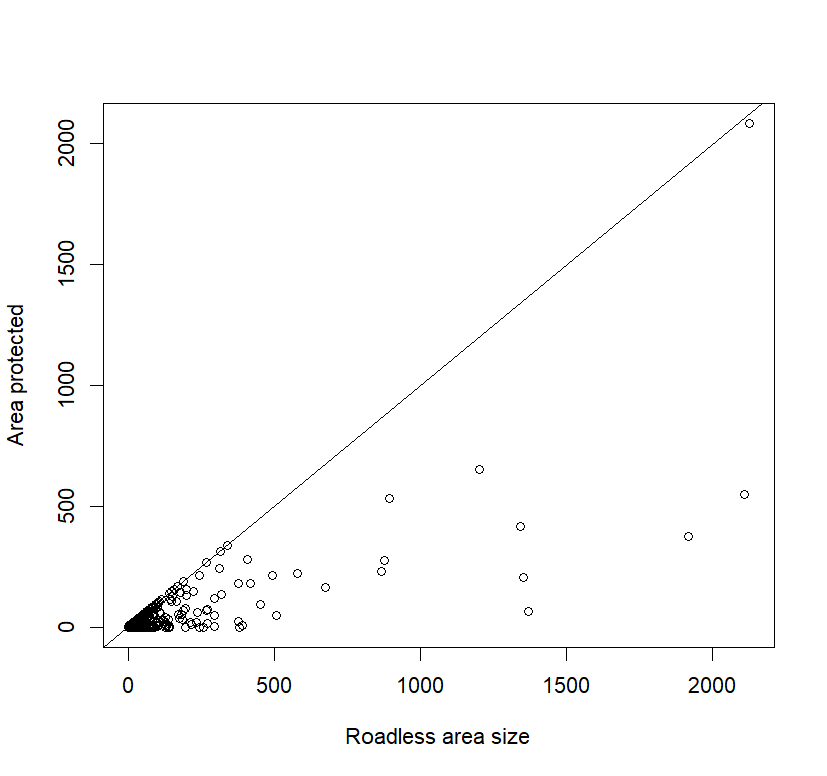


Supplementary Figure 6: Protected area coverage of roadless patches of different size. The relationship between roadless patch size (in km^2^) and the area (in km^2^) within each roadless area that fell under protected areas. The solid line indicates where a roadless patch is 100% protected.

**References**

Arnold, J., Soulsbury, C. D., & Harris, S. 2011. Spatial and behavioral changes by red foxes (*Vulpes vulpes*) in response to artificial territory intrusion. *Canadian Journal of Zoology* 89(9): 808-815

Birks J.D.S. & Kitchener A.C. 1999. The distribution and status of the polecat *Mustela putorius* in Britain in the 1990s. The Vincent Wildlife Trust, London

Chapman, N.G., Claydon, K., Claydon, M., Forde, P.G. & Harris, S. 1993. Sympatric populations of muntjac (*Muntiacus reevesi*) and roe deer (*Capreolus capreolus*): a comparative analysis of their ranging behaviour, social organization and activity. *Journal of Zoology* 229(4): 623-640

Daniels, M.J., Lees, J.D., Hutchings, M.R. & Greig, A. 2003. The ranging behaviour and habitat use of rabbits on farmland and their potential role in the epidemiology of paratuberculosis. *The Veterinary Journal* 165(3): 248-257

Durbin, L.S. 1996. Individual differences in spatial utilization of a river-system by otters *Lutra lutra*. *Acta Theriologica* 41(2): 137-147

Durbin, L.S. 2006. Habitat selection by five otters *Lutra lutra* in rivers of northern Scotland. *Journal of Zoology* 245(1): 85-92

Ferretti, M., Paci, G., Porrini, S., Galardi, L. & Bagliacca, M. 2010. Habitat use and home range traits of resident and relocated hares (*Lepus europaeus*, Pallas). *Italian Journal of Animal Science* 9(3): e54

Gaughran, A. et al. 2018. Super-ranging: A new ranging strategy in European badgers. *PLoS One* 13(2): e0191818

Gazzard, A., Yarnell, R.W. & Baker, P.J. 2022. Fine-scale habitat selection of a small mammalian urban adapter: the West European hedgehog (*Erinaceus europaeus*). *Mammalian Biology* 102: 387-403

Guillet, C., Bergström, R. & Cederlund, G. 1996. Size of winter home range of roe deer *Capreolus capreolus* in two forest areas with artificial feeding in Sweden. *Wildlife Biology* 2(2): 107-111

Harrington, L.A. & Macdonald, D.W. 2008. Spatial and temporal relationships between invasive American mink and native European polecats in the southern United Kingdom. *Journal of Mammalogy* 89(4): 991-1000

Harris, S. 1980. Home ranges and patterns of distribution of foxes (*Vulpes vulpes*) in an urban area, as revealed by radio tracking. *A handbook on biotelemetry and radio tracking*: 685-690

Hofer, H. 1986. Patterns of resource distribution and exploitation by the red fox (*Vulpes vulpes*) and the Eurasian badger (*Meles meles*): a comparative study. University of Oxford.

Hulbert, I.A.R., Iason, G.R., Elston, D.A. & Racey, P.A. 1996. Home-range sizes in a stratified upland landscapes of two lagomorphs with different feeding strategies. *Journal of Applied Ecology* 33(6): 1479-1488

Kenward, R. E. 1982. Techniques for monitoring the behaviour of grey squirrels by radio. *Symposia of the Zoological Society of London* 49: 175-196.

Kolb, H. H. 1984. Factors Affecting the Movements of Dog Foxes in Edinburgh. *Journal of Applied Ecology* 21(1): 161-173.

Kruuk, H. 1978. Spatial organization and territorial behaviour of the European badger *Meles meles*. *Journal of Zoology* 184: 1-19

Kruuk, H.H. and Parish. T. 1987. Changes in the size of groups and ranges of the European badger (*Meles meles* L.) in an area in Scotland. *Journal of Animal Ecology* 56(1): 351-364

Kunst, P.J.G., van der Wal, R. & van Wieren, S. 2001. Home ranges of brown hares in a natural salt marsh: comparisons with agricultural systems. *Acta Theriologica* 46: 287-294

Magowan, E.A. et al. 2022. Dead-reckoning elucidates fine-scale habitat use by European badgers *Meles meles*. *Animal Biotelemetry* 10: 10

McCullough, D.R., Pei, K.C.J. & Wang, Y. 2000. Home range, activity patterns, and habitat relations of Reeves’ muntjacs in Taiwan. *The Journal of Wildlife Management* 64(2): 430-441

Misiorowska, M. 2013. Annual and seasonal home range and distances of movements of released hares (*Lepus europaeus* Pallas, 1778) in Central Poland. *Folia Zoologica* 62(2): 133-142

Morellet, N. et al. 2013. Seasonality, weather and climate affect home range size in roe deer across a wide latitudinal gradient within Europe. *Journal of Animal Ecology* 82(6): 1326-1339

Morris, P. 2009. A study of home range and movements in the hedgehog (*Erinaceus europaeus*). *Journal of Zoology* 214(3): 433-449

Newman, T. J., Baker, P. J., Simcock, E., Saunders, G., White, P. C. L., & Harris, S. 2003. Changes in red fox habitat preference and rest site fidelity following a disease-induced population decline. *Acta Theriologica* 48(1): 79-91

Ó Néill, L., Veldhuizen, T., de Jongh, A. & Rochford, K. 2009. Ranging behaviour and socio-biology of Eurasian otters (*Lutra lutra*) on lowland mesotrophic river systems. *European Journal of Wildlife Research* 55: 363-370

Pandini, W. & Cesaris, C. 1997. Home range and habitat use of roe deer (*Capreolus capreolus*) reared in captivity and released in the wild. *Hystrix Italian Journal of Mammalogy* 9(1-2)

Pettett, C.E., Moorhouse, T.P., Johnson, P.J. & Macdonald, D.W. 2017. Factors affecting hedgehog (*Erinaceus europaeus*) attraction to rural villages in arable landscapes. *European Journal of Wildlife Research* 63: 54

Porteus, T.A., Short, M.J., Hoodless, A.N. & Reynolds, J.C. 2024. Movement ecology and minimum density estimates of red foxes in wet grassland habitats used by breeding wading birds. *European Journal of Wildlife Research* 70: 8

Redpath, S.H.A. et al. 2023. Impact of test, vaccinate and remove protocol on home ranges and nightly movements of badgers in a medium density population. *Scientific Reports* 13: 2592

Reynolds, J. C., & Tapper, S. C. 1995 The ecology of the red fox *Vulpes vulpes* in relation to small game in rural southern England. Wildlife Biology 1: 105-117.

Rossi, I., Lamberti, P., Mauri, L. & Apollonio, M. 2003. Home range dynamics of male roe deer *Capreolus capreolus* in a mountainous habitat. *Acta Theriologica* 48: 425-432

Saïd, S. & Servanty, S. 2005. The influence of landscape structure on female roe deer home-range size. *Landscape Ecology* 20: 1003-1012

Saïd, S. et al. 2009. What shapes intra-specific variation in home range size? A case study of female roe deer. *Oikos* 118: 1299-1306

Saunders, G., White, C. L., Harris, S., Rayner, M. V. 1993. Urban foxes (*Vulpes vulpes*): food acquisition, time and energy budgeting of a generalized predator. *Symposia of the Zoological Society of London* 65: 215-234.

Smith, R.K., Jennings, N.V., Robinson, A. & Harris, S. 2004. Conservation of European hares *Lepus europaeus* in Britain: is increasing habitat heterogeneity in farmland the answer? *Journal of Applied Ecology* 41(6): 1092-1102

Tolhurst, B., Grogan, A., Hughes, H., & Scott, D. 2016. Effects of temporary captivity on ranging behaviour in urban red foxes (*Vulpes vulpes*). *Applied Animal Behaviour Science* 181: 182-190.

Tounzen, M.R., Epperson, D. & Taulman, J.F. 2013. Home range and habitat selection of Eastern gray squirrels (*Sciurus carolinensis*) in a small urban hardwood forest. *Transactios of the Kansas Academy of Science* 115(3&4): 89-101

Voigt, D. R., & Macdonald, D. W. 1984. Variation in the spatial and social behaviour of the red fox, *Vulpes vulpes. Acta Zoologica Fennica* 171: 261-265

White, P. C. L., Saunders, G., & Harris, S. 1996. Spatio-temporal patterns of home range use by foxes (*Vulpes vulpes*) in urban environments. *Journal of Animal Ecology* 65(1): 121-125

Woollard, T., & Harris, S. 1990. A Behavioural Comparison of Dispersing and Non-Dispersing Foxes (*Vulpes vulpes*) and an Evaluation of Some Dispersal Hypotheses. *Journal of Animal Ecology* 59(2): 709-722.
